# Assessing the Enzymatic Hydrolysis of Salmon Frame Proteins through Different By-Product/Water Ratios and pH Regimes

**DOI:** 10.3390/foods10123045

**Published:** 2021-12-08

**Authors:** Pedro Valencia, Silvana Valdivia, Suleivys Nuñez, Reza Ovissipour, Marlene Pinto, Cristian Ramirez, Alvaro Perez, Manuel Ruz, Paula Garcia, Paula Jimenez, Sergio Almonacid

**Affiliations:** 1 Chemical and Environmental Engineering Department, Universidad Técnica Federico Santa María, Valparaíso 2390123, Chile; silvana.valdivia.12@sansano.usm.cl (S.V.); suleivys.nunez@sansano.usm.cl (S.N.); marlene.pinto@usm.cl (M.P.); cristian.ramirez@usm.cl (C.R.); sergio.almonacid@usm.cl (S.A.); 2 Virginia Seafood Agricultural Research and Extension Center, Virginia Tech, Hampton, VA 23669, USA; ovissi@vt.edu; 3 Department of Nutrition, Faculty of Medicine, Universidad de Chile, Santiago 8380453, Chile; afperezb@gmail.com (A.P.); mruz@med.uchile.cl (M.R.); paulagarcia@med.uchile.cl (P.G.); paulajimenez@med.uchile.cl (P.J.); 4 Centro Regional de Estudios en Alimentos Saludables (CREAS), Valparaíso 3100000, Chile

**Keywords:** protein hydrolysis, enzymatic hydrolysis, fish by-product, high by-product concentration

## Abstract

The enzymatic hydrolysis of fish by-product proteins is traditionally carried out by mixing ground by-products with water. In addition, pH control is used to avoid pH drops. Higher costs are involved due to the use of pH control systems and the consequent energy cost in the drying stage. This work aimed to evaluate the effect of these conditions on the hydrolysis of salmon frame (SF) proteins, including the SF hydrolysis without added water. SF hydrolysis by subtilisin at 50, 75, and 100% SF under different pH regimes were evaluated by released α-amino (α-NH) groups, total nitrogen, degree of hydrolysis, and estimated peptide chain length (*PCL*) at 55 °C. The concentration of released α-NH groups was higher in the conditions with less added water. However, the nitrogen recovery decreased from 50 to 24% at 50 and 100% SF, respectively. Changing the SF/water ratio had a more significant effect than changing the pH regime. Estimated *PCL* changed from 5–7 to 7–9 at 50 and 100% SF, respectively. The operating conditions affected the hydrolysis performance and the molecular characteristics of the hydrolysate.

## 1. Introduction

More than 50% of fishery products are discarded as wastes [1,2]. The use of fish by-products involves using zero-cost raw materials that can be converted into low-market-value products, such as meals for animal nutrition or fertilizers, or into high-market-value products, such as functional and bioactive protein hydrolysates for human foods. The fish by-products, which include head, skin, frames, and viscera, are rich sources of proteins, such as collagen. Salmon frames (SF) are the leftovers from salmon fillet production. SF contain 9–15% of the total salmon weight and consist of proteins, lipids, and bones [3]. Compared to the chemical hydrolysis, the enzymatic hydrolysis of fish by-products is an efficient valorization alternative that converts the original proteins into peptides exhibiting various functional properties [4,5]. The enzymatic hydrolysis of SF proteins exhibits high-quality peptides [3], high essential amino acid content [6], and relevant bioactive properties [6,7,8,9]. SF hydrolysates have been tested as food supplements in formulations such as wheat crackers [10] and biscuits [11].

The enzymatic hydrolysis of fish proteins involves a series of unitary operations, as follows: (i) raw-material homogenization, (ii) enzymatic reaction, (iii) inactivation/termination of hydrolysis, and (iv) separation/dehydration [12]. The first step is performed by mixing a certain amount of raw material with water to allow the mixture to be homogenized and, later, agitated during the enzymatic reaction step. Once the reaction stops, the water content is eliminated to obtain a protein hydrolysate in powder format. Technically, the water, which was added in the first step, should be removed in the last step. Thus, the first goal of this study was to compare the performance of SF hydrolysis with and without added water. The novelty of our study is the enzymatic hydrolysis of SF in the condition without added water, the main challenge of which is to keep the system properly mixed. However, as the fish raw material has a high-water content (approximately 60%), it seems plausible to proceed with the enzymatic hydrolysis. Benjakul and Morrisey [13] observed that an increase in the by-product/water ratio results in an increase in α-amino groups released and nitrogen recovery [13]. Vega and Brennan [14] showed that the hydrolysis of cod offal by papain does not require extra water. Results indicated that hydrolysis performance and reaction kinetics are not affected by the high viscosity of the reaction mixture nor by the slow stirring rate [14]. The effect of the SF/water ratio on the hydrolysis performance has been studied by some authors [6,15,16]. In these and other works, the authors used different by-product/water proportions, such as 1:1–1:3 solid/liquid ratio [6], 1:1 salmon co-products/water ratio [8,9], 0.71–1.21 frames/water ratio [15], and 1:10 SF/water (*w*/*v*) ratio [16]. The hydrolysis of SF proteins without added water has not been tested yet (100% SF). The increase in the by-product concentration in the reaction mixture will reduce the drying cost because this is the most expensive stage of hydrolysates production [17]. In addition, in many enzymatic hydrolysis processes, NaOH has been used frequently to maintain the pH at the optimum levels for catalytic activity of the enzyme. The comparison of controlled and uncontrolled pH regimes has not been studied before. The second goal of this study was comparing uncontrolled with controlled pH conditions in terms of reaction efficiency. A quantitative comparison of the enzymatic hydrolysis at different water contents, including the condition without added water, and in different pH control regimes was made in this work. The objective was to evaluate the performance of the SF protein hydrolysis, involving no ideal but economically convenient conditions. The condition without added water and uncontrolled pH corresponds to the main novelty of this study.

## 2. Materials and Methods

### 2.1. Materials

SF were kindly donated by the Group Fiordo Austral, located in southern Chile. Frozen SF were delivered overnight and processed immediately upon arrival. Subtilisin was obtained from the commercial preparation Alcalase 2.4 L, an endoproteinase from Bacillus lichenoformis supplied by Novozymes (Bagsvaerd, Denmark). Analytical-grade reagents were used in all experiments.

### 2.2. Hydrolysis Reaction

Semifrozen SF were ground in a Talsa PSV C15 cutter (Valencia, Spain). Ground SF was stored in 1 kg bags and frozen until use. The hydrolysis reactions were carried out in a vessel containing 450 g of reaction mixture consisting of 50, 75, and 100% (*w*/*w*) of ground SF mixed with water. The vessel with the reaction mixture was kept agitating in a water bath at 55 °C. Three pH regimes were selected: R1, pH 8 controlled; R2, initial pH 8 and uncontrolled; and R3, native pH 6.4 and uncontrolled. Once the temperature and pH were set, subtilisin was added at 13 Anson units (AU) per kg of ground SF. Thus, the subtilisin amount used in each experiment depends on the amount of SF, as described in Table 1, where the resulting subtilisin dose is shown in ppm (mg of subtilisin per kg of reaction mixture). Different subtilisin/SF ratios at 6, 13, and 20 AU/kg were used to evaluate the effect of the enzyme dose at a fixed 100% SF under the pH regime R3. The controlled pH condition was set by a pH-stat method using a Mettler–Toledo T50 autotitrator, where 1 N NaOH was used to neutralize protons. Samples were withdrawn at 0, 1, 2, 5, 10, 20, 30, 40, 50, and 60 min. The experimental conditions are presented in Table 1.

### 2.3. Characterization of Hydrolysis

The total nitrogen contained in SF was quantified by the Kjeldahl method. Withdrawn samples were immediately mixed with an equal volume of 10% trichloroacetic acid and centrifuged at 10,000× *g* × 5 min. Aliquots of this supernatant were used to analyze free and total α-amino (α-NH) groups in the soluble phase. The α-amino groups were quantified by the o-phthalaldehyde method (OPA) [18]. After 60 min of hydrolysis, the reaction mixture was sieved through a fourfold gauze to retain the bones. The fluid phase was centrifuged at 10,000× *g* for 10 min to separate the oil, the soluble phase, and the insoluble phase (pellet). Each phase was weighed. The released water was estimated from the weighed soluble phase. Total α-NH groups were quantified by the OPA method after total hydrolysis in 6 N HCl for 24 h. The total number of α-NH groups was equivalent to the total amount of nitrogen. Nitrogen recovery (*NR*) consisted of the nitrogen transferred from salmon frames to the soluble phase, according to Equation (1).

(1) $$NR\left(\%\right)=\frac{total\;nitrogen\;in\;soluble\;phase\left(mmoles\right)}{total\;nitrogen\;in\;reaction\;mixture\left(mmoles\right)}\times 100,$$

The specific yield ($Y_{sp}$) was defined as the amount of nitrogen per kg of salmon and protease amount in *AU* after 60 min of hydrolysis, according to Equation (2).

(2) $$Y_{sp}=\frac{total\;nitrogen\;in\;soluble\;phase\left(mmoles\right)}{mass\;of\;salmon\;frames\left(kg\right)\times protease\left(AU\right)},$$

The degree of hydrolysis (*DH*) was calculated by an approximation (*DH*’) from the soluble α-NH groups/total nitrogen ratio. The *DH* determination according to Benjakul and Morrisey [13] was not technically feasible with this heterogenous raw material because of the presence of bones. Therefore, the α-NH in the original by-product (*L*_0_ according to Ref. [13]) was not quantified. In consequence, the total peptide bonds were slightly overestimated from the total nitrogen, resulting in a slight underestimation of the *DH*, according to Equation (3).

(3) $$DH^{’}\left(\%\right)=\frac{free\;\alpha \text{-}NH\;groups\;in\;soluble\;phase\left(mmoles\right)}{total\;nitrogen\;in\;reaction\;mixture\left(mmoles\right)}\times 100,$$

The estimation of the peptide chain length (*PCL*) was calculated by the ratio of total nitrogen to total free α-NH groups after 60 min of hydrolysis, according to Equation (4).

(4) $$PCL=\frac{total\;nitrogen\;in\;soluble\;phase\left(mmoles\right)}{free\;\alpha \text{-}NH\;groups\;in\;soluble\;phase\left(mmoles\right)},$$

*PCL* is inversely related to the *DH*, as reviewed by Kristinsson and Rasco [1]. All the hydrolysis experiments were carried out in duplicate. The plotted data are the mean of two experimental points, and the error bars are their standard deviation. Sample analyses were carried out in triplicate. Correlations were analyzed by Pearson’s R and the probability (*p*) from ANOVA.

## 3. Results

**Figure 1:**
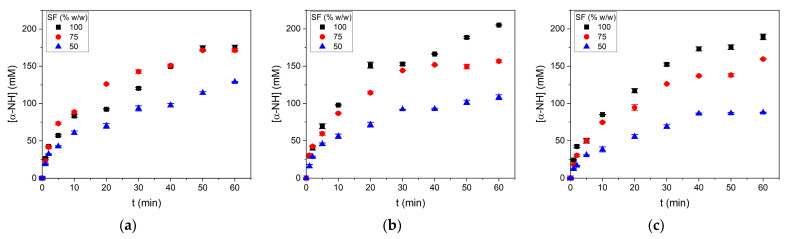
Reaction progress of α-NH group concentration during the hydrolysis of SF in different SF/water mixtures under different pH regimes. (**a**) R1: controlled pH 8; (**b**) R2: initial pH 8 uncontrolled; (**c**) R3: initial pH 6.4 uncontrolled. Reaction conditions were 55 °C and 13 AU/kg of SF. Each point is the mean of two experimental points, and the error bars are the standard deviation.

Ground SF samples were hydrolyzed by subtilisin in different SF/water mixtures under different pH regimes in an agitated batch reactor. Pictures of reaction mixtures can be observed in Supplementary Material. The results of the reaction progress are shown in Figure 1.

In general, the concentration of released α-NH groups was higher in the conditions with less added water. The impact generated by the SF/water ratio is more significant than the effect of pH regimes. This is a significant finding considering that the protease subtilisin expresses more activity in alkaline pH. The initial and final pH values obtained in the experiments are shown in Table 2. In the uncontrolled pH condition R3, the reaction began at pH 6.4–6.5 (native pH) and dropped by 0.3 pH units after 60 min of reaction. However, under the pH regime R2, the pH dropped by between 1.2 and 1.4 pH units from the initial pH 8. The pH drop depended on the SF proportion and the initial pH value. Higher SF/water ratios and a lower initial pH were correlated to lower pH drops. A higher protein concentration in the reaction mixture buffered the pH during the reaction progress.

**Figure 2:**
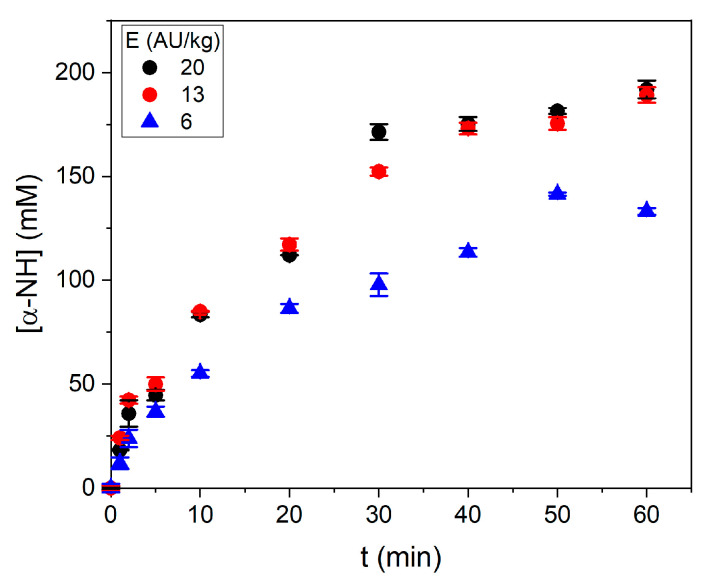
Progress of hydrolysis at different protease doses. The reaction conditions were 100% SF, pH regime R3, and 55 °C. Each point is the mean of two experimental points, and the error bars are the standard deviation.

The effect of the protease dose was evaluated at 100% SF and 55 °C under the pH regime R3, which corresponds to the combination of both non-ideal conditions tested in this work. The results are shown in Figure 2. Three protease doses were tested: 6, 13, and 20 AU per kg of SF. The results indicated that an increase in the protease dose from 6 to 13 AU/kg increased the hydrolysis efficiency. However, a new increase, from 13 to 20 AU/kg, did not cause an increase in reaction efficiency. The decision of using 13 AU of subtilisin per kg of SF was based on this result. This dose of subtilisin concords with the study of Liaset et al. [15], where E/S ratios between 30 and 90 AU of Protamex per kg of crude protein were used. Considering that the protein content was 16.2% of crude protein in SF, they used between 4.7 and 14.6 AU of Protamex per kg of SF. In other published works, Idowu et al. [16] used between 8.6 and 26.0 AU of Alcalase per kg of SF, while He et al. [8] used between 12 and 72 AU per kg of salmon co-products. In all the cases, the protease dose was within the same range of Anson units used in our study. Differences in reaction performance could depend on the protein content of the SF or co-products.

A saturation of cleavage sites on the superficial area of SF particles could explain the lack of increase in reaction efficiency when using more than 13 AU/kg of SF. This is a major concern because protease additions over 13 AU/kg do not increase the reaction efficiency. In addition, since protease is the highest cost issue during hydrolysis operation, this is a key point to focus on in a cost-efficiency analysis of the process. The adsorption of subtilisin on the protein was suggested by O’Meara and Munro [19] during a study of lean meat protein by Alcalase.

**Figure 3:**
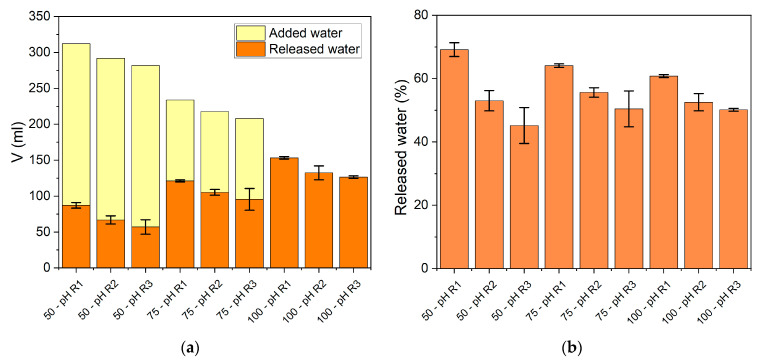
Volumes and percentages of water released during the hydrolysis of SF proteins after 60 min of reaction in different SF/water mixtures under different pH regimes. (**a**) Added and released water for all experimental conditions; (**b**) percentage of water released with respect to the SF water content. The reaction conditions were 55 °C and 13 AU/kg of SF. Each point is the mean of two experimental points, and the error bars are the standard deviation.

A high SF concentration was feasible and convenient in terms of the high concentration of α-NH groups released, the buffering of pH drop, and the lowest amount of water. Nevertheless, knowing the complexity of the enzymatic hydrolysis of proteins, a deep insight is needed. A wide set of parameters were evaluated when the reaction stopped. These included the released water from the SF tissue, the α-NH group yield, the total nitrogen released, nitrogen recovery (NR), the degree of hydrolysis (*DH*’), and peptide size estimation. The necessity of adding water has been based on improving mixing during the reaction. However, considering that the water content of SF is 53%, the amount of water in the reaction mixture is half of the by-product mass in the 100% m/m SF condition. In our experiments, the agitation never presented a challenge because even with 100% m/m SF, the reaction mixture could be mixed and behaved as a viscous fluid. After the addition of protease, the mixture evolved to a fluid suspension during the first minute of reaction (data not shown). The water contained in SF was released during the hydrolysis of proteins due to the degradation of large proteins. The amounts of water involved in hydrolysis reactions are plotted in Figure 3. In general, around 50% of the water contained in SF was released after 60 min of the hydrolysis reaction. The amount of released water was affected by both the SF proportion and the pH regime. The higher SF proportions and the more alkaline pH conditions generated higher water release. However, although higher amounts of water were released, it corresponded to a lower percentage of the total water contained in SF tissue (Figure 3b). The pH regimes R2 and R3 achieved more similar percentages of released water, around 50%.

**Figure 4:**
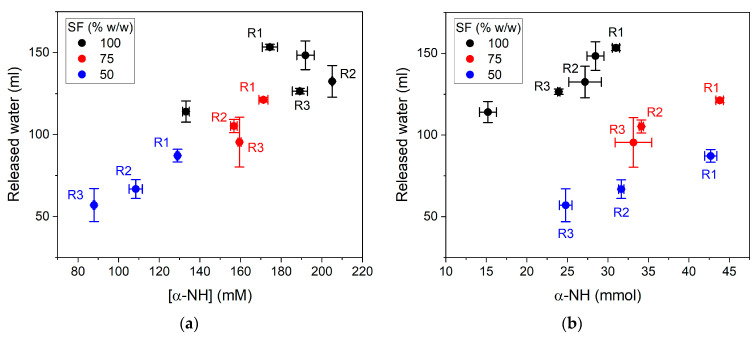
Correlation between released water and α-NH groups during the hydrolysis of SF proteins after 60 min of reaction at 55 °C with different protease doses; SF/water mixtures; and pH regimes R1, R2, and R3, according to Table 1. (**a**) Released water versus the α-NH group concentration; R = 0.884, *p* = 3.02 × 10^−4^; (**b**) released water versus the number of α-NH groups; R = 0.998, *p* = 0.041 at 50% SF; R = 0.954, *p* = 0.19 at 75% SF; and R = 0.929, *p* = 0.022 at 100% SF. Each point is the mean of two experimental points, and the error bars are the standard deviation.

The results of all experiments were plotted to observe the correlation of released water with the concentration and number of α-NH groups in Figure 4. High concentrations of α-NH groups did not mean a high number of α-NH groups (mmoles in Figure 4b). The experiments with different protease doses than 13 AU/kg were included. The amount of released water was interestingly correlated with the concentration of α-NH groups, independently of the reaction conditions, with Pearson’s R = 0.884. The correlation depended on the reaction conditions in the case of the number of α-NH groups (Figure 4b). Pearson’s R was 0.998, 0.954, and 0.929 for 50, 75, and 100% SF conditions, respectively. It is well established that water and protein are associated at the molecular level. Thus, the release of α-NH groups from SF proteins inevitably involves the release of the linked water and its transfer, along with peptides, toward the soluble phase. As mentioned above, the number of released α-NH groups was not necessarily higher in the more concentrated SF reactions. Some batches in the 50 and 75% SF condition overmatched the number of α-NH groups released in the 100% SF condition. However, as the amount of added and released water was higher in the 50 and 75% SF conditions, the resulting concentrations of α-NH groups were lower than that in the 100% SF. A high number of α-NH groups and a low water volume are convenient results. However, the number of α-NH groups corresponds to an estimation of the peptide concentration in the soluble phase and it does not consider the size of the peptides.

**Figure 5:**
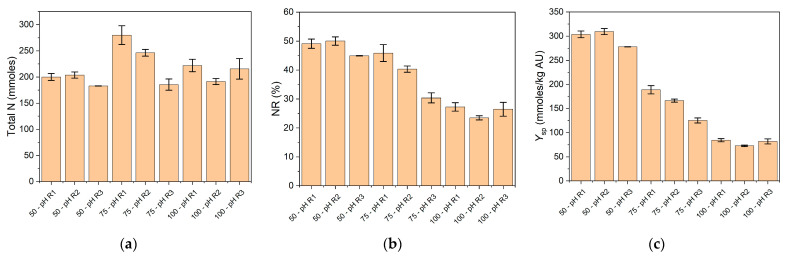
Nitrogen extraction and specific yield obtained after 60 min of SF hydrolysis from different SF/water mixtures under different pH regimes. (**a**) Total amount of nitrogen in the soluble phase; (**b**) nitrogen recovery: percentage of SF nitrogen transferred to the soluble phase; (**c**) nitrogen-specific yield: amount of nitrogen transferred to the soluble phase per mass of SF and protease amount. Reaction conditions were 55 °C and 13 AU/kg of SF. Each point is the mean of two experimental points, and the error bars are the standard deviation.

Another parameter considered to evaluate the reaction productivity is the amount of nitrogen extracted from the SF and transferred to the soluble phase. This value was used to calculate the nitrogen recovery (NR), according to Equation (1), and the specific yield of nitrogen, according to Equation (2). These parameters were plotted in Figure 5.

**Figure 6:**
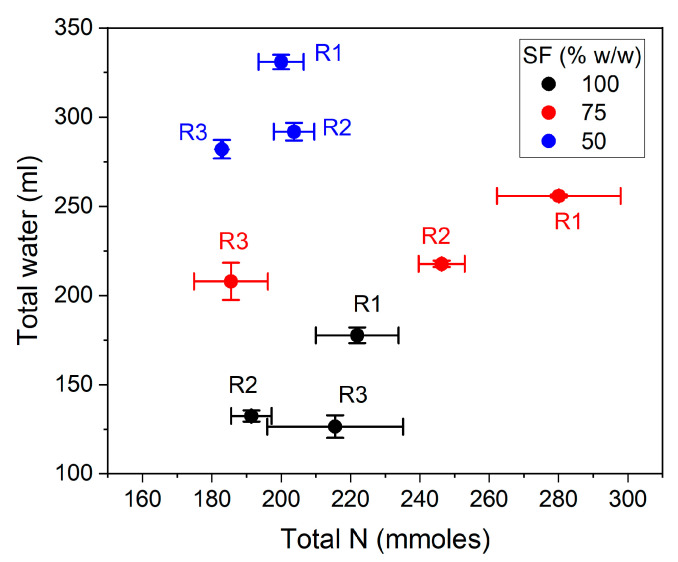
Total water (released + added) versus total nitrogen obtained after 60 min of SF hydrolysis in different SF/water mixtures under different pH regimes R1, R2, and R3, according to Table 1. The reaction conditions were 55 °C and 13 AU/kg of SF. Each point is the mean of two experimental points, and the error bars are the standard deviation.

The total nitrogen amount transferred to the soluble phase was around 200 mmoles in the different reaction conditions except for the 75% SF under pH regimes R1 and R2 (Figure 5a). These conditions achieved 280 and 246 mmoles of total nitrogen extracted in the soluble phase, respectively. The profile of total nitrogen through the different reaction conditions was not clear. However, the percentage of nitrogen extracted was clearly decreased when SF increased (Figure 5b). A more dramatic profile was obtained with the nitrogen-specific yield plotted against the different reaction conditions. The highest specific yield was 300 mmoles/kg·AU, while the lowest value was 73 mmoles/kg·AU, for the 50 and 100% SF, respectively. The most convenient condition can be established by aiming to obtain the highest NR, the lowest volume of water, and the lowest protease dose. A plot of total water versus total nitrogen is presented in Figure 6. The total water obtained after the SF hydrolysis was not correlated with the total nitrogen transferred to the soluble phase (no statistical analysis) and just depended on the amount of added water. The total nitrogen transferred to the soluble phase was between 183 and 280 mmoles and did not correlate with the percentage of SF in the reaction mixture. The 100% SF batch will achieve the lowest cost of water evaporation during the drying stage. Twice the mass of water and, of course, drying cost is obtained in the 50% SF condition. Thus, the economic convenience will be between the 75 and 100% SF conditions depending on the gain from the nitrogen obtained and the cost of the drying process. However, these high-SF conditions involve high protease doses, which should be included in the economic evaluation.

**Figure 7:**
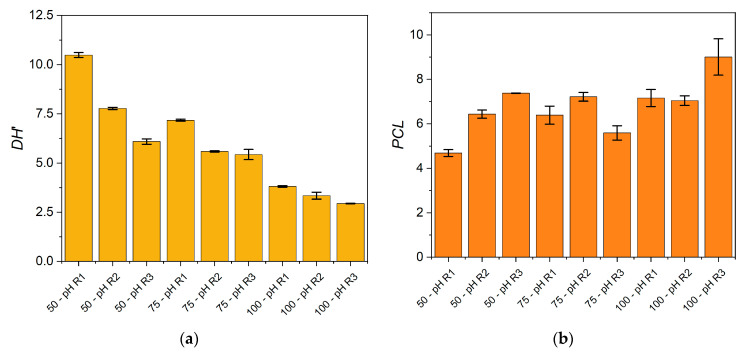
Characterization of the hydrolysates in the soluble phase after 60 min of reaction at 55 °C with different protease doses, SF/water mixtures, and pH regimes. (**a**) Estimated degree of hydrolysis (*DH*’); (**b**) estimated peptide chain length (*PCL*). Each point is the mean of two experimental points, and the error bars are the standard deviation.

In addition to the process characterization, the degree of hydrolysis (*DH*) and the peptide chain length (*PCL*) were determined for each condition. The *DH* was estimated from the free α-NH groups/total nitrogen ratio considering that it should be calculated from the α-NH groups/total peptide bonds ratio. Thus, an underestimation of the *DH* was obtained and denominated *DH*’. The *PCL* corresponds to a characterization of peptides through the calculation of the total nitrogen/free α-NH groups ratio, both quantified in the soluble phase. The *DH*’ and *PCL* obtained in each reaction condition were plotted in Figure 7.

The results showed a decrease of the *DH*’ when increasing the SF/water ratio and when uncontrolled pH regimes were applied. The effect on *PCL* was exactly contrary due to the inverse relationship between *DH*’ and *PCL* (Equations (3) and (4)). Larger peptides were produced at higher SF/water ratios under uncontrolled pH regimes. We can infer that the addition of water to the reaction mixture is a modulating parameter to modify the characteristics of the protein hydrolysate. As *DH*’ and *PCL* are molecular characteristics that modulate hydrolysates’ functional properties, we have found that different operating conditions will generate different hydrolysates in terms of these properties. Thus, the desired hydrolysate properties can be aimed through the manipulation of the reaction conditions.

## 4. Discussion

The study of the by-product/water ratio effect on the hydrolysis performance has been assessed in some publications. However, the hydrolysis of fish by-products without added water has been only published by Vega and Brennan (1988) [14]. This is, to the best of our knowledge, the only previous research on this issue. Our findings agree with that publication in terms that the agitation and mixing in the batch reactor are feasible and, definitively, not a problem at all. The viscosity of the ground SF is not enough to impede proper mixing. Furthermore, the hydrolysis of large proteins decreases rapidly and enormously the initial agitation resistance. We can declare that the addition of water is no longer an argument to increase the mixing properties of fish by-product hydrolysis. This clearly decreases the energy costs during the drying stage. The effect of water addition was not only limited to the mixing properties but also affected the reaction performance and hydrolysates properties by changing the by-product concentration and the volume of the soluble phase. The main effects observed among the different reaction conditions considering the SF/water ratio and pH regimes were in terms of the number and concentration of α-NH groups released. The higher the SF/water ratio, the higher the concentration of α-NH groups released. However, these higher concentrations did not mean a higher number of α-NH groups or higher total nitrogen extracted. The protease subtilisin was chosen because it is the most cost efficient compared with other commercial and extracted proteases [20]. The results showed lower subtilisin activity under the pH regime R3 compared to that in regimes R1 and R2. It was evidenced in the lower concentrations (Figure 4a) and the number of α-NH groups released (Figure 4b). However, the activity was good enough to produce a similar number of α-NH groups with controlled pH as with the uncontrolled pH regimes (Figure 4b). Despite these findings, new studies are needed to evaluate the hydrolysis performance using neutral proteases according to the pH of the ground SF (pH 6.4–6.5).

At this point, an economic analysis is needed to establish which condition is the most convenient, considering that higher SF/water ratios and uncontrolled pH regimes are low-cost but less productive. The reaction conditions studied also affected the characteristics of the hydrolysates, which is a significant concern in terms of functional properties. The *DH*’ and *PCL* were affected by the SF/water ratios and the pH regimes. We observed that the larger peptide sizes were obtained at higher SF/water ratios under uncontrolled pH regimes. We can infer that the proportion between water and by-product affected the protease distribution between both phases, the surface of by-product particles (muscle and bones) and the soluble phase, containing hydrolyzed protein and peptides. According to this hypothesis, the catalytic action of protease would be distributed as an adsorbed protease and a free protease; thus, the cleavage of peptide bonds will occur on the surface of particulate material and the already hydrolyzed peptides dissolved in the soluble phase. The predominating catalytic action will depend on the distribution of the protease molecules. We have now formulated the hypothesis that the protease distribution can be modulated by the operating conditions. In addition, this modulation will affect the molecular size of peptides and their functional properties. Future research aims to apply the knowledge obtained in this study to improve the hydrolysis performance and to evaluate the protease distribution in both the particulate material (insoluble phase) and the soluble aqueous phase. Furthermore, an economic evaluation of the process can be made. The operating costs at different SF/water ratios under different pH regimes can be evaluated considering the drying stage and its associated energy costs.

## 5. Conclusions

A novel study of the SF protein hydrolysis has been performed in non-ideal conditions such as 100% SF in the reaction mixture (without added water) and uncontrolled pH. The hydrolysis of SF proteins by subtilisin was technically feasible without both the addition of water and pH control. However, the nitrogen recovery decreases at higher SF proportions (less added water), but this may still be convenient because of the savings in the drying stage. The effect of different SF/water ratios evidenced an effect on the hydrolysis performance and on the molecular size of peptides in the hydrolysate. We postulate that a lower-water condition promotes a preferent distribution of the protease on the particulate material than in the soluble phase, generating a hydrolysate with a higher proportion of large peptides. Therefore, the functional properties and, consequently, the product’s added value, could be modulated by the operating conditions.

## Figures and Tables

**Table 1:** Experimental conditions for the hydrolysis of SF proteins in a 450 g reaction mixture at 55 °C under different pH regimes.

SF (% *w*/*w*)	SF Mass (g)	Subtilisin/SF Ratio (AU/kg)	Subtilisin Dose (ppm)	pH Regimes
50	225.0	13	13.6	R1, R2, R3
75	337.5	13	20.3	R1, R2, R3
100	450.0	13	27.1	R1, R2, R3
100	450.0	6	12.5	R3
100	450.0	20	41.7	R3

**Table 2:** Initial and final pH values for each experimental replicate after 60 min of SF hydrolysis at 55 °C and 13 AU/kg.

pH Regime	pH Value	SF % (*w*/*w*)					
		50		75		100	
		1	2	1	2	1	2
R1	Initial	8.054	7.918	8.193	7.997	7.962	8.041
	Final	7.996	7.994	8.060	8.000	7.997	7.996
R2	Initial	8.060	7.990	8.040	8.030	7.940	7.890
	Final	6.730	6.630	6.660	6.670	6.790	6.720
R3	Initial	6.497	6.465	6.454	6.414	6.405	6.430
	Final	6.171	6.161	6.136	6.099	6.125	6.140

## Supplementary Materials

The following are available online at https://www.mdpi.com/article/10.3390/foods10123045/s1, Pictures of reaction mixtures before and after hydrolysis of SF by subtilisin, separated oil and soluble phase, and centrifuged mixture containing insoluble phase, soluble phase and oil.

## References

[1] Kristinsson H.G., Rasco B.A. (2000). Fish protein hydrolysates: Production, biochemical, and functional properties. Crit. Rev. Food Sci. Nutr..

[2] Chalamaiah M., Dinesh Kumar B., Hemalatha R., Jyothirmayi T. (2012). Fish protein hydrolysates: Proximate composition, amino acid composition, antioxidant activities and applications: A review. Food Chem..

[3] Liaset B., Julshamn K., Espe M. (2003). Chemical composition and theoretical nutritional evaluation of the produced fractions from enzymic hydrolysis of salmon frames with Protamex™. Process Biochem..

[4] Halim N.R.A., Yusof H.M., Sarbon N.M. (2016). Functional and bioactive properties of fish protein hydolysates and peptides: A comprehensive review. Trends Food Sci. Technol..

[5] Najafian L., Babji A.S. (2012). A review of fish-derived antioxidant and antimicrobial peptides: Their production, assessment, and applications. Peptides.

[6] Vázquez J.A., Sotelo C.G., Sanz N., Pérez-Martín R.I., Rodríguez-Amado I., Valcarcel J. (2019). Valorization of Aquaculture By-Products of Salmonids to Produce Enzymatic Hydrolysates: Process Optimization, Chemical Characterization and Evaluation of Bioactives. Mar. Drugs.

[7] Idowu A.T., Igiehon O.O., Idowu S., Olatunde O.O., Benjakul S. (2021). Bioactivity Potentials and General Applications of Fish Protein Hydrolysates. Int. J. Pept. Res. Ther..

[8] He S., Franco C., Zhang W. (2012). Process optimisation and physicochemical characterisation of enzymatic hydrolysates of proteins from co-products of Atlantic Salmon (*Salmo salar*) and Yellowtail Kingfish (*Seriola lalandi*). Int. J. Food Sci. Technol..

[9] Slizyte R., Rommi K., Mozuraityte R., Eck P., Five K., Rustad T. (2016). Bioactivities of fish protein hydrolysates from defatted salmon backbones. Biotechnol. Rep..

[10] Idowu A.T., Benjakul S., Sinthusamran S., Pongsetkul J., Sae-Leaw T., Sookchoo P. (2019). Whole wheat cracker fortified with biocalcium and protein hydrolysate powders from salmon frame: Characteristics and nutritional value. Food Qual. Saf..

[11] Singh A., Benjakul S., Huda N. (2020). Characteristics and nutritional value of biscuits fortified with debittered salmon (*Salmo salar*) frame hydrolysate. Int. J. Food Sci. Technol..

[12] Valencia P.L., Flores S.A., Pinto M.J., Almonacid S.F. (2016). Analysis of the operational strategies for the enzymatic hydrolysis of food proteins in batch reactor. J. Food Eng..

[13] Benjakul S., Morrissey M.T. (1997). Protein Hydrolysates from Pacific Whiting Solid Wastes. J. Agric. Food Chem..

[14] Vega R.E., Brennan J.G. (1988). Enzymic hydrolysis of fish offal without added water. J. Food Eng..

[15] Liaset B., Nortvedt R., Lied E., Espe M. (2002). Studies on the nitrogen recovery in enzymic hydrolysis of Atlantic salmon (*Salmo salar*, L.) frames by Protamex™ protease. Process Biochem..

[16] Idowu A.T., Benjakul S., Sinthusamran S., Sookchoo P., Kishimura H. (2019). Protein hydrolysate from salmon frames: Production, characteristics and antioxidative activity. J. Food Biochem..

[17] Himonides A.T., Taylor A.K.D., Morris A.J. (2011). Enzymatic Hydrolysis of Fish Frames Using Pilot Plant Scale Systems. Food Nutr. Sci..

[18] Nielsen P.M., Petersen D., Dambmann C. (2001). Improved Method for Determining Food Protein Degree of Hydrolysis. J. Food Sci..

[19] O’Meara G.M., Munro P.A. (1985). Kinetics of the hydrolysis of lean meat protein by alcalase: Derivation of two alternative rate equations and their fit to experimental data. Biotechnol. Bioeng..

[20] Kristinsson H.G., Rasco B.A. (2000). Kinetics of the hydrolysis of Atlantic salmon (*Salmo salar*) muscle proteins by alkaline proteases and a visceral serine protease mixture. Process Biochem..

